# Low-dose oral prednisone improves clinical and ultrasonographic remission rates in early rheumatoid arthritis: results of a 12-month open-label randomised study

**DOI:** 10.1186/ar3838

**Published:** 2012-05-14

**Authors:** Carlomaurizio Montecucco, Monica Todoerti, Garifallia Sakellariou, Carlo Alberto Scirè, Roberto Caporali

**Affiliations:** 1 Division of Rheumatology, IRCCS Policlinico S. Matteo Foundation, University of Pavia, Piazzale Golgi 2, 27100 Pavia, Italy

## Abstract

**Introduction:**

In early rheumatoid arthritis (RA), low-dose oral prednisone (PDN) co-medication yields better clinical results than monotherapy with disease-modifying anti-rheumatic drugs (DMARDs). In addition, ultrasonography (US) evaluation reveals rapid and significant effects of glucocorticosteroids on subclinical synovitis. No data currently exist that examine the clinical and US results offered by glucocorticoid co-medication over DMARD monotherapy in early RA patients.

**Methods:**

Two hundred and twenty patients with early RA (< 1 year from clinical onset) were treated according to a low disease activity (LDA) targeted step-up protocol including methotrexate (MTX) and, in the active treatment arm, low-dose (6.25 mg/day) oral PDN over 12 months. Clinical disease activity measures were collected at baseline, 2, 4, 6, 9 and 12 months, and US examination of hands was performed at baseline, 6 and 12 months. Grey-scale and power Doppler (PD) synovitis were scored (0 to 3) for each joint. At 12 months, clinical remission according to the disease activity score among 28 joints was defined as the clinical outcome, and a total joint PD score of 0 (PD negativity) as the imaging outcome.

**Results:**

Each group included 110 patients with comparable demographic, clinical, laboratory and US characteristics. At 12 months, the LDA rate was similar in the two groups, whilst the clinical remission rate (risk ratio = 1.61 (95% confidence interval = 1.08, 2.04)) and PD negativity rate (risk ratio = 1.31 (95% confidence interval = 1.04, 1.64)) were significantly higher in the MTX+PDN group.

**Conclusion:**

In early RA, despite a similar response rate in terms of LDA, low-dose oral PDN co-medication led to a higher proportion of clinical remission and PD negativity compared with MTX monotherapy, thus ensuring a better disease activity control.

**Trial registration number:**

Current Controlled Trials ISRCTN2486111

## Introduction

Glucocorticoids (GC) in association with conventional disease-modifying anti-rheumatic drugs (DMARDs) are currently recommended for the treatment of rheumatoid arthritis (RA), after a careful evaluation of the patient's risk-to-benefit ratio [1].

In RA, GC show a rapid symptomatic effect on pain and general well-being and help in controlling objective signs of inflammation, such as tender and swollen joints [2-4]. Several studies also support disease-modifying properties for GC, in terms of prevention of future joint damage in early RA, both for initial short-term high dosages followed by step-down schedules and for low-dose oral therapy regimens [3-8].

On the whole, in clinical trials involving early-onset RA patients treated with low-dose oral GC in association with DMARDs, clinical and functional benefits seem to last only few months after the start of treatment, fading away thereafter, whilst prevention of structural damage may persist over a longer time [5,6]. These studies suggest a sort of dissociation between a short-lasting clinical improvement and a long-lasting effect on radiographic progression [9]. Whether the effect on structural damage is just related to a faster clinical response or may be associated with a persistently deeper control of joint inflammation, not fully detectable by clinical tools, is still to be ascertained.

Sensitive imaging techniques may help in testing these hypotheses. Recent imaging studies on RA have demonstrated that the structural progression observed in patients in clinical remission might be explained by the persistence of subclinical signs of synovitis detected by magnetic resonance imaging or ultrasonography (US) [10]. In particular, power Doppler (PD) US has been demonstrated to be a valid and reliable tool in detecting subclinical inflammation in RA, and is believed to be a measure of activity of joint inflammation [11,12]. At present there are no data on the effect of low-dose oral GC on US-detected synovial inflammation in early RA.

This study aimed to verify the effect of low dosages of oral prednisone (PDN) on clinical and US outcomes over 12 months of follow-up in patients with recent-onset RA. For this purpose we randomly allocated patients to receive low-dose PDN or not within a structured treat-to-target protocol aiming for low disease activity (LDA) [13]. Disease activity was prospectively assessed both clinically and by US.

## Methods

### Patients

All patients attending the Early Arthritis Clinic of a University Hospital between January 2005 and January 2008, who were part of a larger cohort study, were eligible for this open-label randomised clinical trial.

Inclusion criteria were: fulfilment of the American College of Rheumatology classification criteria for RA, age > 18 years, symptom duration < 12 months, active disease according to the disease activity score [14,15]. Exclusion criteria were: contraindications for steroidal therapy, including uncontrolled diabetes and previous fragility osteoporotic fractures.

The local medical ethics committee approved the study protocol, and all patients gave written informed consent before study inclusion.

### Interventions

All patients in the overall cohort followed a disease-activity-driven step-up therapeutic protocol aimed at reaching a state of LDA according to the disease activity score [15]. In more detail, patients started methotrexate (MTX) at baseline at a dosage of 10 mg/week; if at the following visits they did not reach a LDA state, MTX was increased, if tolerated, to 15 mg/week, then to 20 and 25 mg/week. Patients who did not achieve the target with the maximum tolerated dosage of MTX started anti-TNF therapy in association with the ongoing medication, if not contraindicated.

Given this background therapeutic strategy, patients were randomised to also receive or not low-dose oral PDN, starting with 12.5 mg/day for 2 weeks tapered to 6.25 mg/day for the follow-up period, in a once-a-day morning administration.

Oral folic acid supplementation (5 mg/week) was introduced during treatment with MTX.

During the follow-up, switching to other DMARDs was permitted in cases of intolerance or toxicity. Concomitant therapy with nonsteroidal anti-inflammatory drugs and intra-articular injections with GC when required for refractory monoarticular involvement were allowed. The patients were also given prophylactic treatment with oral calcium and vitamin D when not contraindicated.

### Clinical assessment

Patients were clinically assessed at baseline and every 2 months during the first 6 months, and then every 3 months thereafter. A four-variable disease activity score among 28 joints (DAS28) was computed as the disease activity measure at each visit. Remission according to DAS28 was defined for values < 2.6 [16]. Clinical remission was also assessed by the Simplified Disease Activity Index (SDAI) as recently suggested [17]. For study purposes, clinical evaluation included: number of swollen and tender joints on 28-joint count, and visual analogue scale (VAS) scores for pain, global health assessment and overall disease activity (patient's global assessment); functional disability was also measured by a self-assessment of the Italian version of the Health Assessment Questionnaire [18,19]. The erythrocyte sedimentation rate and C-reactive protein serum levels were measured at each time point.

### Ultrasonographic assessment

US was performed at baseline, 6 and 12 months by a single experienced operator unaware of clinical data using a GE Logiq 9 scanner (General Electrics Medical Systems, Milwaukee, WI, USA) with a multi-frequency linear array transducer (10 to 15 MHz), according to the European League Against Rheumatism guidelines [20].

The US assessment included a limited 12-joint assessment with transverse and longitudinal scanning of the medial and lateral dorsal views of bilateral wrists (radiocarpal, ulnocarpal, radio-ulnar and midcarpal joints) and metacarpophalangeal joints. PD calibrations were adjusted at the lowest permissible pulse repetition frequency to maximise sensitivity and were taken as constant for the same joint in different patients (dependent on the investigated joint), with a pulse repetition frequency ranging from 0.5 to 0.75 MHz. Colour gain was set just below the level that causes the appearance of noise artefacts. Flow was demonstrated in two perpendicular planes and confirmed by the pulsed-wave Doppler spectrum to exclude artefacts [21]. Grey-scale (GS) and PD signals were assigned to each singular joint in accordance with semi-quantitative scales (0 = normal, 1 = mild, 2 = moderate, 3 = severe), as previously reported [22]. Two cumulative US joint scores (GS score and PD score) were calculated at each US assessment as the sum of either GS or PD signal scores obtained from each joint. US remission was defined as a PD score of 0 at 12 months, as recently proposed [23]. Each patient evaluation took 20 minutes on average, and representative images were archived.

### Outcomes

Evaluated primary outcomes included: the proportion of patients achieving clinical remission according to DAS28 (< 2.6) and the proportion of patients achieving PD negativity (defined as a global PD score of 0) at 12 months [23]. The combined clinical and US outcome was also explored, as both DAS28 remission and PD negativity. Finally, clinical remission was also assessed by the SDAI, as recently suggested [17].

### Sample size, randomisation and blinding

The sample size was defined based on a proportion difference of 20% with a power of 0.8, allowing for 10% of attrition. Allocation of patients to treatment was carried out using an independently generated randomisation list.

Although this is an open-label randomised study, US was performed by an ultrasonographer who was blinded to clinical data - including the therapeutic arm - in order to avoid differential misclassification.

### Statistical methods

Intention-to-treat and per-protocol analyses were performed. For intention-to-treat analysis, patients were analysed according to the treatment group to which they were originally assigned when at least one single dose of PDN was taken; while for per-protocol analysis, only patients who continued the initial treatment were effectively analysed. Clinical quantitative variables are reported by the median (interquartile range) or by the mean ± standard deviation, when appropriate. To test differences between the two groups, the Mann-Whitney test or the independent-sample *t *test was used for continuous variables and the Fisher's exact test was used for proportions. As measures of association we used the mean or median difference from baseline (MD) for continuous variables, and risk ratios (RRs) and incident rate ratios for categorical variables. Missing data on outcome were not imputed and complete case analysis was performed in all cases.

All significance tests were two-tailed at the 0.05 significance level. All analyses were conducted using Stata version 11 (StataCorp, College Station, TX, USA).

## Results

### Patients

**Figure 1:**
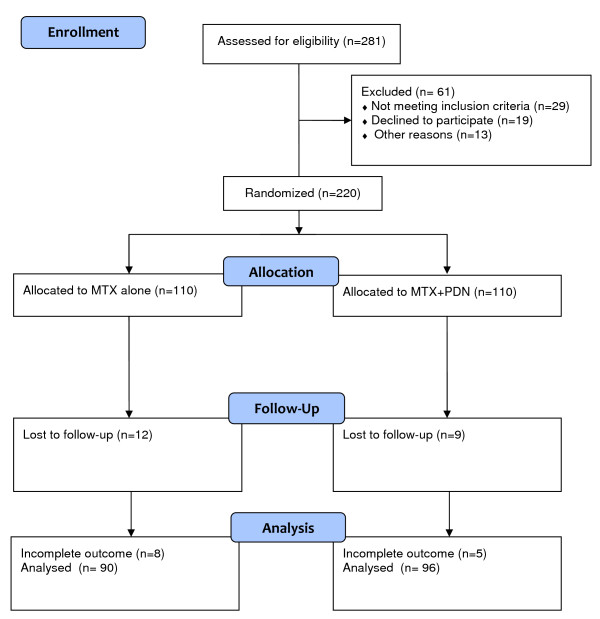
**Flow chart of patients enrolled in the trial**. MTX, methotrexate monotherapy; MTX+PDN, methotrexate + prednisone combination therapy.

A total of 220 patients were enrolled in the study; 110 were randomised to the MTX+PDN group and 110 to MTX monotherapy. The number of patients lost to the follow-up is shown in Figure 1. A total of 186 patients with complete clinical and US follow-up data were included in the analyses: 90 patients in the MTX arm and 96 patients in the MTX+PDN arm. Baseline characteristics of patients included in the analyses did not differ from excluded patients.

At baseline, the subjects assigned to the two therapeutic arms did not significantly differ for demographics, disease activity and disability indexes (Table 1).

**Table 1:** Baseline demographic, clinical, laboratory and ultrasonographic characteristics of patients according to therapeutic arm

	Methotrexate alone	Methotrexate + prednisone	*P *value
*n*	110	110	
Age (years)	62 (51.5 to 72)	57 (45 to 67)	0.06
Sex (female/male)	69/23	71/28	0.60
Symptoms duration (months)	3.48 (2.57 to 5.70)	2.97 (1.93 to 5.10)	0.08
DAS28	5.2 (4.4 to 5.9)	5 (4.2 to 5.9)	0.78
VAS score for pain	51 (46.5 to 79)	55 (40 to 79)	0.64
VAS score for PGA	60 (48 to 75)	60 (47 to 80)	0.61
VAS score for general health status	60 (50 to 72)	56 (50 to 75)	0.92
Swollen joints among 28 joints	8 (4 to 12)	9 (5 to 14)	0.23
Tender joints among 28 joints	8.5 (3.5 to 13)	6 (3 to 12)	0.06
Health Assessment Questionnaire	1 (0.6 to 1.6)	1.1 (0.6 to 1.6)	0.62
Erythrocyte sedimentation rate (mm/hour)	23.5 (12 to 39)	28 (16 to 48)	0.09
C-reactive protein (mg/dl)	0.7 (0.3 to 1.9)	1.2 (0.3 to 2.7)	0.11
US grey scale score	8 (3 to 11)	8 (4 to 12)	0.49
US power Doppler positive	57 (51)	65 (59)	0.27
US power Doppler score	1 (0 to 6)	2 (0 to 7)	0.39

Data presented as median (interquartile range) or n (%). DAS28, disease activity score among 28 joints; VAS, visual analogue scale; PGA, patient's global assessment; US, ultrasonography.

### Treatment

Patients in the MTX+PDN group were less likely to adhere to the protocol, with a RR of 0.82 (95% confidence interval (CI) = 0.69, 0.96). The main reasons for discontinuation in the GC group (15 patients) were mild side effects (six patients) and unwillingness to continue GC therapy (nine patients). Among the 90 completers in the MTX monotherapy group, five patients started GC medication because of increased disease activity over time. The maximum MTX dose was 14.3 ± 4.3 mg/day in the combination therapy group and 16.1 ± 3.9 mg/day in the MTX monotherapy group (*P *= 0.053).

Therapeutic adjustments were made in 101 patients - 57 patients for MTX alone and 44 patients for the MTX+PDN arm - with a trend toward less therapeutic adjustments in the MTX+PDN group (incident rate ratio = 0.77, 95% CI = 0.50, 1.16; *P *= 0.19). Sixteen patients in the MTX group and nine patients in the MTX+PDN group started anti-TNF therapy before the completion of 12 months of follow-up. At the end of the follow-up, six and 10 patients, respectively, were still in treatment with conventional DMARDs despite not achieving LDA; nine patients denied consent for biologic therapy, three patients had intercurrent adverse events leading to delay in treatment steps, and four patients had disease reactivations at the last visit. Eight patients in the MTX arm and six patients in the MTX+PDN arm underwent intra-articular or peri-articular steroid injections.

### Clinical response

At the end of the follow-up period, LDA according to the disease activity score was achieved by 145 out of 186 patients (77.9%), without significant differences between the two treatment arms: 68 patients (75.5%) in the MTX group and 77 patients (80.2%) in the MTX+PDN group (*P *= 0.44).

**Figure 2:**
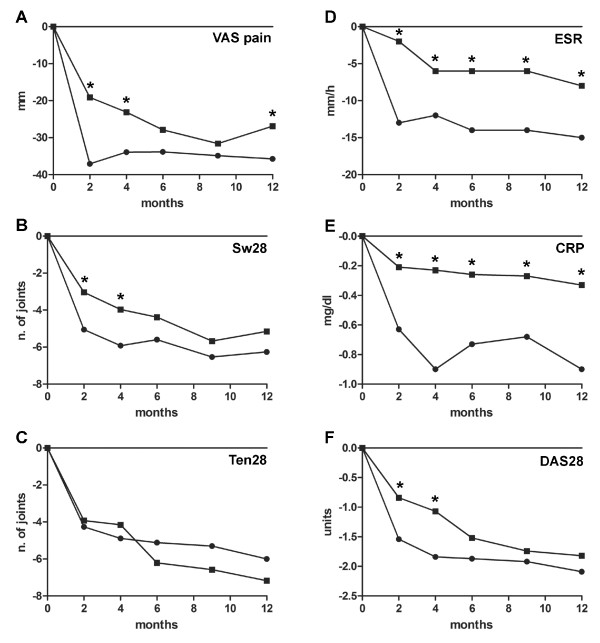
**Trend over time of measures of clinical subjective and objective variables**. Trend over time of measures of clinical subjective and objective variables along the two treatment arms. Subjective: **(A) **visual analogue scale (VAS) score for pain. Objective: **(B) **swollen joint count over 28 joints (Sw28), **(C) **tender joint count over 28 joints (Ten28), **(D) **serum levels of the erythrocyte sedimentation rate (ESR), **(E) **serum levels of C-reactive protein (CRP), and **(F) **absolute mean values of disease activity score among 28 joints (DAS28). *P < 0.05 related to differences between treatment arms.

On the whole, subjective (VAS score for pain, VAS score for patient's global assessment, VAS score for global health assessment) and objective (swollen and tender joint counts) variables, as well as US variables, significantly improved throughout the follow-up (Figure 2A to 2F).

Comparing the two therapeutic arms, the mean VAS score for pain (Figure 2A) fell more rapidly in the MTX+PDN arm, reaching a significant difference between groups at 2 months (MD = -17.9 (95% CI = -26.4, -9.4), *P *< 0.001) and at 4 months (MD = -10.8 (95% CI = -19.1, -2.5), *P *= 0.01). This difference was not significant at 6 and 9 months, while it was weakly significant at the end of the follow-up (MD = -8.8 (95% CI = -17.5, -0.1), *P *= 0.04). Similarly, the mean swollen joint count (Figure 2B) fell more rapidly in the MTX+PDN arm, reaching a significant mean difference at 2 months (MD = -2.0 (95% CI = -3.6,-0.3), *P *= 0.01) and 4 months (MD = -1.9 (95% CI = -3.6, -0.2), *P *= 0.02). This difference was not significant at 6, 9 and 12 months. No significant differences were observed for the tender joint count (Figure 2C) throughout the entire follow-up period. Acute-phase reactants (erythrocyte sedimentation rate and C-reactive protein) showed a significantly greater improvement (Figure 2D,E) in the MTX+PDN group throughout the entire period of follow-up.

Comparing the two therapeutic arms, the mean DAS28 score (Figure 2F) fell more rapidly in the MTX+PDN group, while the differences became not statistically significant throughout the follow-up: the DAS28 MD was -0.69 (95% CI = -1.04, -0.34), *P *< 0.001 at 2 months; -0.76 (95% CI = -1.12, -0.39), *P *< 0.001 at 4 months; -0.35 (95% CI = -0.72, 0.01), *P *= 0.06 at 6 months; -0.17 (95% CI = -0.55, 0.20), *P *= 0.36 at 9 months; and -0.27 (95% CI = -0.67, 0.12), *P *= 0.18 at 12 months.

GS and PD scores also showed a more rapid response in the MTX+PDN group. The GS score MD was -1.78 (95% CI = -3.66, 0.09), *P *=0.06 at 6 months and -1.52 (95% CI = -0.66, 3.71), *P *= 0.17 at 12 months. The PD score MD was -1.72 (95% CI = -3.44, -0.01), *P *= 0.04 at 6 months and -1.24 (95% CI = -3.21, 0.73), *P *= 0.21 at 12 months.

### Outcome analysis

The frequency of patients achieving clinical remission (DAS28 < 2.6) was significantly higher in the MTX+PDN group than in the MTX group: 43/96 (44.8%) versus 25/90 (27.8%) (*P *= 0.02). The relative risk of clinical remission was on average 60% higher in the group of combination therapy when compared with MTX monotherapy (RR = 1.61 (95% CI = 1.08, 2.04)). Globally, SDAI remission was achieved less frequently than DAS28 remission; however, MTX+PDN-treated patients had significantly higher probability of SDAI remission over MTX monotherapy arm at 12 months (30.8% vs. 16%), with a RR of 1.91 (95% CI = 1.08, 3.38; *P *= 0.01).

**Figure 3:**
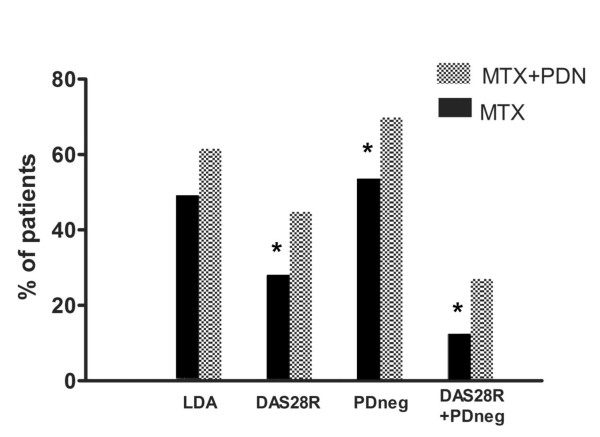
**Patients' relative frequencies of clinical and/or ultrasonographic outcomes according to treatment group**. DAS28R, clinical remission according to the disease activity score among 28 joints; LDA, low disease activity; MTX, methotrexate monotherapy; MTX+PDN, methotrexate + prednisone combination therapy; PDneg, power Doppler signal negativity; DAS28R+PDneg, combined outcome of clinical remission according to disease activity score among 28 joints and power Doppler negativity. *P < 0.05 related to differences between treatment arms.

Similarly, the probability of reaching PD negativity was significantly higher in the MTX+PDN group (67/96, 69.8%) than in the MTX group (48/90, 53.3%) (*P *= 0.04), with a RR at 12 months of 1.31 (95% CI = 1.04, 1.64). The combined clinical and US outcome was achieved by 34/96 patients (35.5%) and 14/90 (15.9%) patients in the MTX+PDN and MTX groups, respectively (*P *= 0.01), with a RR of 2.27 (95% CI = 1.31, 3.95). The abovementioned clinical and imaging outcomes are presented in Figure 3, shown as the percentages of patients reaching the outcomes at the end of the follow-up period.

Per-protocol and intention-to-treat analysis results were almost similar: performance on combination therapy was significantly better over MTX monotherapy for both clinical and US outcomes. Specifically, the frequency of patients achieving clinical remission (DAS28 < 2.6) was significantly higher in the MTX+PDN group than in the MTX group with a relative risk (RR) of 1.65 (95% CI = 1.07, 2.54; *P *= 0.01). Similarly, the probabilities of reaching PD negativity and the combined clinical and US outcome were significantly higher in the MTX+PDN group than in the MTX group: RR = 1.34 (95% CI = 0.99, 1.81; *P *= 0.05) and RR = 2.49 (95% CI = 1.27, 4.88; *P *= 0.005), respectively.

### Adverse events

Six adverse events related to GC medication were reported in six patients; namely, hyperglycaemia (two patients) and epigastric pain (four patients). Side effects related to MTX medication - such as nausea, fatigue or slight increase in liver test function - leading to discontinuation were found in 10 patients from the MTX group and in six patients in the PDN co-medication group (*P *= 0.29).

## Discussion

Our data indicate that low-dose oral PDN leads to higher probability of clinical remission and PD negativity at 1 year of follow-up in a LDA-driven therapeutic protocol in early-onset RA patients. We found that the percentage of subjects achieving LDA at 1 year was not statistically different between the two treatment arms. This is not surprising with a treat-to-target therapeutic approach along with a higher frequency of therapeutic adjustments in the MTX monotherapy group. Nevertheless, GC co-medication gave an earlier and faster clinical response in terms of DAS28 values over time, with significant intergroup difference already detectable after 2 months of treatment, persisting up to 6 months.

The prompt clinical effect of GC co-medication in early RA has already been reported by several authors [2-5]. In this regard, Kirwan presented no sustained clinical advantage on GC co-medication in early RA patients since the addition of prednisolone to DMARDs accelerated, but did not enhance, the clinical response [2]. The clinical benefits on singular clinical parameters under a very low dose (5 mg/day) of GC co-medication also did not continue beyond the first year of follow up in Wassenberg and colleagues' early RA cohort [5]. Only Svensson and colleagues found a sustained clinical effect on low-dose oral prednisolone (7.5 mg/day) as an additional therapy to DMARDs in early RA patients [7].

Analysing in our study the trend of each singular clinical and laboratory parameter over time, we observed only an initial substantial advantage in adding GC compared with MTX monotherapy in clinical variables, with no persistent differences. Most of the benefits associated with GC co-medication on DAS28 response seems to be explained by significantly greater, faster and persistent control over time on acute-phase reactant serum levels (erythrocyte sedimentation rate and C-reactive protein). This might also have indirect relevant implications, considering their prognostic value, especially for C-reactive protein levels over time, in predicting subsequent structural damage both in early and established RA [24,25].

The analysis of the US data strengthens this result. There is evidence that a subgroup of RA patients with drug-induced sustained remission still have detectable active subclinical synovitis [10,23]. We analysed the percentage of PD negativity in early-onset disease after achieving DAS28 remission. The analysis revealed that PD negativity was almost 1.5-fold higher in the PDN co-medication group than in the MTX monotherapy group (Additional file 1 shows this in more detail).

Previous studies on the effect of steroid medication on US measures in RA are scanty and mostly refer to the favourable short-term effect of a single intravenous, intra-articular or high-dose oral administration in established disease [26-29].

In our study, median US score values significantly decreased during follow-up in the whole series of patients, but GC-treated patients presented a trend toward a greater and more rapid response, so that GC-treated patients more frequently reached a completely negative PD status at the end of follow-up. Nevertheless, in both treatment arms, PD score trends over 6 months of follow-up were not predictive of the subsequent achievement of clinical remission. This finding is particularly important due to the predictive role of PD active synovitis on relevant disease outcomes [10,22]. As the persistency of a positive PD signal is the main predictor of early relapse and radiographic progression in patients in clinical remission, the higher effect of GC on PD may explain the long-term beneficial effect on structural damage.

The particular setting of an inceptional cohort enabled us to analyse the specific effect of a structured therapeutic protocol including or not including low-dose PDN in untreated patients from the onset of the disease. Furthermore, this study is the first controlled clinical trial exploring the effect of low-dose oral GC on US outcomes in early RA. The blinded assessment of US outcome strengthens the validity of these results.

The present study has some limitations. The sample size was able to detect only large differences between the two therapeutic arms, so the study was underpowered for subgroup analyses - such as the specific effect of GC on the patients achieving clinical remission. Furthermore, caution should be taken when interpreting the positive results in terms of the combined clinical and US variables, as this combined outcome was not prespecified. A reduced number of joints (*n *= 12) was assessed by US in our study. Although a similar US evaluation has been extensively applied and validated in RA, we are aware that a small percentage of joints with signs of active synovitis might be lost in this way [30-32]. Moreover, the therapeutic strategy applied in our study is less aggressive than currently proposed MTX escalation schedules [33,34]. This characteristic is related to the time when this study was designed and might have increased the relative effect of combination therapy over MTX monotherapy. Finally, the sample size and length of follow-up did not allow us to measure the effect of GC on rare events, such as the appearance of new radiographic erosions or some side effects such as osteoporotic fractures. In relation to the safety profile, data from our analyses described few relevant side effects related to low-dose PDN medication, in accordance with a recent meta-analysis presenting a very good safety profile [35,36]. In general, patients on GC were more likely to withdraw the treatment protocol due to unwillingness to proceed with GC medication, as also reported in a previous cross-sectional survey [37].

## Conclusion

Our results suggest that in patients with early-onset RA, the addition of low-dose oral PDN to a MTX background in the contest of a treat-to-target strategy may ensure a faster and better control of disease activity, with higher rates of both clinical remission and PD signal negativity at 1 year.

To our knowledge, this study shows for the first time that a greater clinical and subclinical effect of low-dose GC co-medication over DMARD monotherapy was demonstrated in early RA patients in a controlled manner. Further data on subsequent structural damage might confirm and clarify the role of such deeper control of inflammation on GC therapy. The acceptable safety profile along with the positive clinical and imaging results should allow us to suggest such a treatment strategy in the management of early disease.

## Abbreviations

DAS28: disease activity score among 28 joints; DMARD: disease-modifying anti-rheumatic drug; GC: glucocorticoids; GS: grey-scale; LDA: low disease activity; MD: mean or median difference from baseline; MTX: methotrexate; PD: power Doppler; PDN: prednisone; RA: rheumatoid arthritis; RR: risk ratio; SDAI: Simplified Disease Activity Index; TNF: tumour necrosis factor; US: ultrasonography; VAS: visual analogue scale.

## Competing interests

The authors declare that they have no competing interests.

## Authors' contributions

All authors were involved in drafting or substantially revising the article, and all authors approved the final version to be published. CM and RC were responsible for study conception and design. MT, GS and CAS were responsible for acquisition of data. CM, RC, CAS, GS and MT were responsible for analysis and interpretation of data.

## Supplementary Material

Additional file 1 **Figure showing ultrasound images in JPEG format**. Ultrasound images at start of treatment (left side) and after 1 year of follow-up (right side) in the two treatment arms (MTX alone, first line; MTX+PDN, second line). RA patients on combination therapy with low-dose oral prednisone achieved PD negativity significantly more than MTX monotherapy treated patients at 1 year of follow up. Click here for file
