# Supplementary figures and images for: Low-dose oral prednisone improves clinical and ultrasonographic remission rates in early rheumatoid arthritis: results of a 12-month open-label randomised study
Source: Arthritis Res Ther. 2012 May 14;14(3):R112. doi: 10.1186/ar3838 (PMC3446489; doi:10.1186/ar3838)

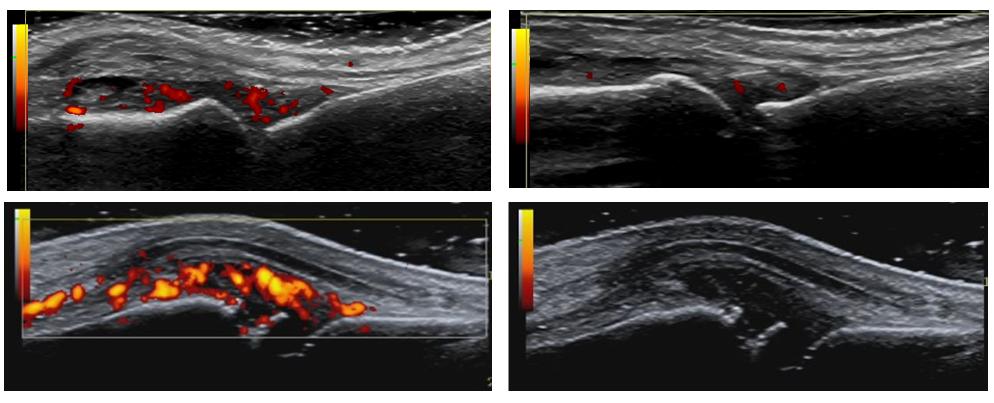

Supplement: Additional file 1 — Figure showing ultrasound images in JPEG format. Ultrasound images at start of treatment (left side) and after 1 year of follow-up (right side) in the two treatment arms (MTX alone, first line; MTX+PDN, second line). RA patients on combination therapy with low-dose oral prednisone achieved PD negativity significantly more than MTX monotherapy treated patients at 1 year of follow up. [file ar3838-S1.JPEG]
